# Long-term no-till: A major driver of fungal communities in dryland wheat cropping systems

**DOI:** 10.1371/journal.pone.0184611

**Published:** 2017-09-12

**Authors:** Dipak Sharma-Poudyal, Daniel Schlatter, Chuntao Yin, Scot Hulbert, Timothy Paulitz

**Affiliations:** 1 Oregon Department of Agriculture, Salem, Oregon, United States of America; 2 United States Department of Agriculture-Agricultural Research Service, Wheat Health, Genetics and Quality Research Unit, Pullman, Washington, United States of America; 3 Department of Plant Pathology, Washington State University, Pullman, Washington, United States of America; Universita degli Studi di Pisa, ITALY

## Abstract

In the dryland Pacific Northwest wheat cropping systems, no-till is becoming more prevalent as a way to reduce soil erosion and fuel inputs. Tillage can have a profound effect on microbial communities and soilborne fungal pathogens, such as *Rhizoctonia*. We compared the fungal communities in long-term no-till (NT) plots adjacent to conventionally tilled (CT) plots, over three years at two locations in Washington state and one location in Idaho, US. We used pyrosequencing of the fungal ITS gene and identified 422 OTUs after rarefication. Fungal richness was higher in NT compared to CT, in two of the locations. *Humicola nigrescens*, *Cryptococcus terreus*, *Cadophora* spp. Hydnodontaceae spp., and *Exophiala* spp. were more abundant in NT, while species of *Glarea*, *Coniochaetales*, *Mycosphaerella tassiana*, *Cryptococcus bhutanensis*, *Chaetomium perlucidum*, and *Ulocladium chartarum* were more abundant in CT in most locations. Other abundant groups that did not show any trends were *Fusarium*, *Mortierella*, *Penicillium*, *Aspergillus*, and *Macroventuria*. Plant pathogens such as *Rhizoctonia* (Ceratobasidiaceae) were not abundant enough to see tillage differences, but *Microdochium bolleyi*, a weak root pathogen, was more abundant in NT. Our results suggest that NT fungi are better adapted at utilizing intact, decaying roots as a food source and may exist as root endophytes. CT fungi can utilize mature plant residues that are turned into the soil with tillage as pioneer colonizers, and then produce large numbers of conidia. But a larger proportion of the fungal community is not affected by tillage and may be niche generalists.

## Introduction

Since the dawn of agriculture, tillage has played an important role by maintaining a disturbance that favors domesticated plants [1]. Disturbance by mechanical implements is used to prepare and cultivate the seed-bed, control weeds, break up residue, and incorporate manures and fertilizer. However, tillage can have a detrimental effect on soil sustainability by reducing organic matter through exposure to oxygen and microbial decomposition, breaking up soil aggregates and fungal networks [2, 3], but most importantly by increasing soil erosion from water and wind. The rate of soil loss from agriculture far exceeds the rate of soil formation [4]. In 1997, an estimated 2.0 billion tons of soil were lost in the US [5]. One way to reduce soil loss is to stop tillage by using no-till methods. This practice has increased significantly in the US over the last 30 years, especially in soybean, corn, and wheat cropping systems. Overall in the US in 2012, 96 million acres were planted with no-till out of 278 million total acres under cultivation [6]. In reduced tillage or no-till (NT), also called direct-seeding, seeds are planted directly into the residue of the previous crop with minimal disturbance of the residue [7]. Besides reducing soil erosion, no-till can conserve soil moisture, increase water infiltration, increase soil organic matter, and result in increased earthworm populations [8].

Tillage can also have a profound effect on soil microbial communities, by breaking up soil aggregates, exposing microsites to oxygen, and increasing microbial activity. Studies of the bacterial component of soil communities have demonstrated significant impacts of tillage on bacterial community composition and microbial activity [9, 10], though others have found little or no significant impact of tillage [11–14]. The literature on effects of tillage on the arbuscular mycorrhizal fungi (AMF) is more consistent. Numerous studies show either detrimental effects or shifts in the communities of AMF, where diversity, richness, and abundance of AMF increased in no-till or reduced tillage systems [11, 15–18]. However, most of the literature on AMF or other fungal groups is based on phospholipid fatty acid (PLFA) or fatty acid methyl esterase (FAME), which quantifies broad groups of microbes based on molecules in their membranes. These methods do not have the resolution to delineate fungal community composition in detail. In the early 2000s, more researchers used PCR amplification, cloning and sequencing of ribosomal genes that have taxonomic applications in fungi- internal transcribed spacer (ITS) and large ribosomal subunit (LSU). However, these methods are still limited by the size of the library of clones that that can be made, usually just a few hundred [19]. The use of next-generation sequencing (NGS) offers the ability to examine fungal communities in far greater detail in agricultural ecosystems, especially the effect of tillage.

Fungi play a major role in no-till systems in terms of carbon cycling, soil health, and disease or disease suppression. In the dryland wheat cropping systems of the Pacific Northwest (PNW), Rhizoctonia bare patch and root rot can increase significantly when tillage is stopped, during the conversion from conventional to no-till or direct seeding [20, 21]. It often takes two years for the disease to increase, but once it does, yield is significantly reduced. However, in long-term no-till systems, *Rhizoctonia* is not a problem, based on a comparison of no-till fields to adjacent conventionally-tilled fields [22]. Could shifts in the microbial community be responsible for suppression of *Rhizoctonia*? Bacterial communities can be affected by tillage in the PNW [23], but tillage had a much weaker effect on bacterial communities compared to proximity to root or location. The bacterial communities in the rhizosphere which would have the most profound effect on root rotting fungi, were more buffered from the effects of tillage than those in the bulk soil. Very few bacterial taxa were consistently shown to be affected by tillage. What about fungal communities? In Australia, fungi were implicated in the suppression of *Rhizoctonia* in a long-term no-till system [24]. Fungi are the primary decomposers of plant residue and play an important role in the carbon cycle. They also produce hyphal networks in the soil that may be sensitive to disruption by conventional tillage practices. Because of the profound effect of tillage on residue decomposition, we hypothesize that fungal communities will be significantly affected in terms of diversity, richness and composition. More specifically, we hypothesize that certain taxa will be favored by lack of tillage (NT) and others will be more predominant in conventional tillage (CT). To address these hypotheses, we sampled plots that had been in long-term no-till (12–32 years), and adjacent plots that were in long-term conventional tillage. This was done at two dryland wheat locations in eastern Washington and one location in northern Idaho, with samples taken over three years.

## Materials and methods

### Survey sites and tillage treatments

Three experimental sites included in the study were in the Palouse region of Idaho and Washington. The Palouse region has Palouse silt loam soils with an average pH of 5.6, 1.78% organic C, and 0.12% total N within the top 10 cm [25]. One location was in Idaho (Kambitsch Farm) and two locations were in Washington (USDA-ARS Palouse Conservation Farm, PCFS, and R. J. Cook Agronomy Farm, or the Cook Farm). The Kambitsch Farm, Cook Farm, and PCFS have been managed and maintained by University of Idaho, Moscow, ID; Washington State University, Pullman, WA; and USDA-ARS, Pullman, WA, respectively.

The Kambitsch Farm is located at north of Genesee, ID (46°35'17.0"N 116°56'49.8"W). This farm had five replicated NT and CT treatment plots. The crop rotation was winter wheat-spring wheat or spring barley-grain legume (pea, lentil or chickpea). The Cook Farm, Pullman, WA (46°47'00.6"N 117°04'40.6"W) had long-term direct seed cropping systems research program. This experimental site had no-till treatment for 13 years. Adjacent to the NT block, had a conventional tillage block. Conventional tillage was done with chisel plows. Since this site did not have replicated treatment plots, four directional quadrants (NE, NW, SE, and SW) were considered as replications for each treatment (CT vs NT). PCFS site (46°45'30.0"N 117°11'35.4"W) had two treatments, conventional tillage and no-till management for 35 years. There were 4 replications. The crop rotation was winter wheat/spring wheat in the no-till plot. The conventional tillage plot with a winter wheat/fallow rotation was adjacent to each no-till plot. Soil was fall chiseled, spring-disked, and rod weeded 3 to 5 times during the fallow years. In all plots, the winter wheat part of the rotation was sampled.

### Soil sampling and DNA extraction

Soil samples were collected in 2012, 2013, and 2014 at Kambitsch Farm and PCFS sites whereas samples were collected from Cook Farm in 2013 and 2014. Bulk soil consisted of three sub-samples collected randomly from each plot. Crop residue on the soil surface was removed and soil was collected from the top 20 cm at the tillering stage of wheat and from other plots simultaneously. Each sub-sample was approximately 1 kg. Each sample was sieved through a 2-mm mesh to eliminate crop residues and debris. Three sub-samples from each plot were composited and homogenized. Soil samples were stored in 50 ml plastic tubes (Thermo Fisher Scientific, MA), stored at -20°C and thawed just before DNA extraction. Total DNA was extracted from 0.5 g of each soil sample using UltraClean Soil DNA Kit (MO BIO Laboratories, CA) as described previously [26] and stored at -20°C for subsequent procedures.

### Sequencing of the Fungal ITS region

A total of 83 DNA samples (23 samples collected in 2012, 30 in 2013, and 30 in 2014) was used for NGS (454 pyrosequencing). The ITS 1–4 region was amplified using fungi specific primers (ITS1-F: 5’-CTTGGTCATTTAGAGGAAGTAA-3’; ITS4: 5’-TCCTCCGCTTATTGATATGC-3’[27] as per MrDNA (Shallowater, TX) protocols. Amplicons were sequenced using a Roche 454 FLX titanium instruments and with reagents following the manufacturer’s guidelines. Raw sequence data has been submitted to the NCBI Sequence Read Archive under the study accession #SRP114697.

### DNA sequence processing

Raw 454 flowgrams were trimmed to 450 flows and denoised in MOTHUR v1.36.1 [28] using shhh.seqs. Barcodes and primers were trimmed and any sequence with a homopolymer >8bp or <200bp in length were discarded. Denoised sequences from both runs were combined and chimeric sequences were identified with the usearch61 algorithm implemented in identify_chimeric_seqs.py in QIIME v1.9.1 [29]. Open-reference OTU picking was performed with pick_open_reference_otus.py using the UNITEv7 dynamic (31.01.16) reference set using a 97% similarity threshold. Taxonomy was assigned to OTUs by blasting representative sequences against the UNITE general release (31.01.16). To ensure that only high-quality OTUs were included, those with low e-values (<10e-40), low match length to representative sequences (query length/subject length<0.75), or <10 total sequences were discarded. After processing, sequencing yielded an average of 7,562 sequences/sample (+/- 2023), with a minimum of 2157 and a maximum of 15,524. To ensure equal sampling depth, samples were rarefied to 3,679 sequences/sample prior to analyses and samples (n = 1) with less than this number of sequences were discarded.

### Statistical analyses

The composition of fungal communities was evaluated using non-metric multidimensional scaling (NMDS) plots of Bray-Curtis distances using the metaMDS function of the vegan package in R [30, 31]. The significance of different factors to fungal community structure was assessed using PERMANOVA implemented in the adonis function in vegan with 1000 permutations to determine significance. Fungal richness, the inverse Simpson’s diversity index (1/D), and the Shannon diversity index (S’) were calculated using vegan and compared using a 3-way ANOVA with year, location, and tillage as factors. The Simpson’s index is more weighted toward community evenness, while the Shannon index is weighted towards community richness. Proportions of fungal phyla were compared across tillage treatments, locations, and years using Kruskal-Wallis tests. Relative abundances of abundant fungal general and OTUs (genera <0.5% total relative abundance or OTUs with >200 total sequence counts) were compared with ANOVA after log10(1+x) transformation of sequence counts using a Benjamini-Hochberg correction for false discovery rates.

## Results

### Fungal community composition

After sequence processing and quality filtering, 626,182 sequences belonging to 987 fungal OTUs remained (297,999 sequences among 422 OTUs after rarefaction). Fungal communities were dominated by members of the Ascomycota (~82.5% ± 9.6% of all sequences; S1 Fig) followed by the Basidiomycota (~12.5% ± 8.7% of all sequences; S1 Fig). There was a significantly greater proportion of Basidiomycetes in no-till fields versus conventionally-tilled (NT: 15.4 ± 4.6%; CT: 7.7 ± 10.5%; Kruskal-Wallis test p = 0.001) sites, and significantly small proportion of Ascomycetes (79.2% ± 10.9% in NT, 86 ± 0.06% in CT; Kruskal-Wallis test p = 0.002). The relative abundances of these phyla did not differ significantly among locations (Kruskal-Wallis test: Ascomycota *χ*^2^ = 2.69, p = 0.26; Basidiomycota *χ*^2^ = 2.92, p = 0.23) or years (Kruskal-Wallis test: Ascomycota *χ*^2^ = 4.78, p = 0.091; Basidiomycota *χ*^2^ = 1.73, p = 0.42). Zygomycota, Chytridiomycota, and unidentified phyla composed smaller proportions of communities that did not differ significantly among tillage treatments (S1 Fig; Kruskal-Wallis test p>0.62), though relative abundances of these minor phyla differed significantly among locations and years (data not shown).

**Fig 1:**
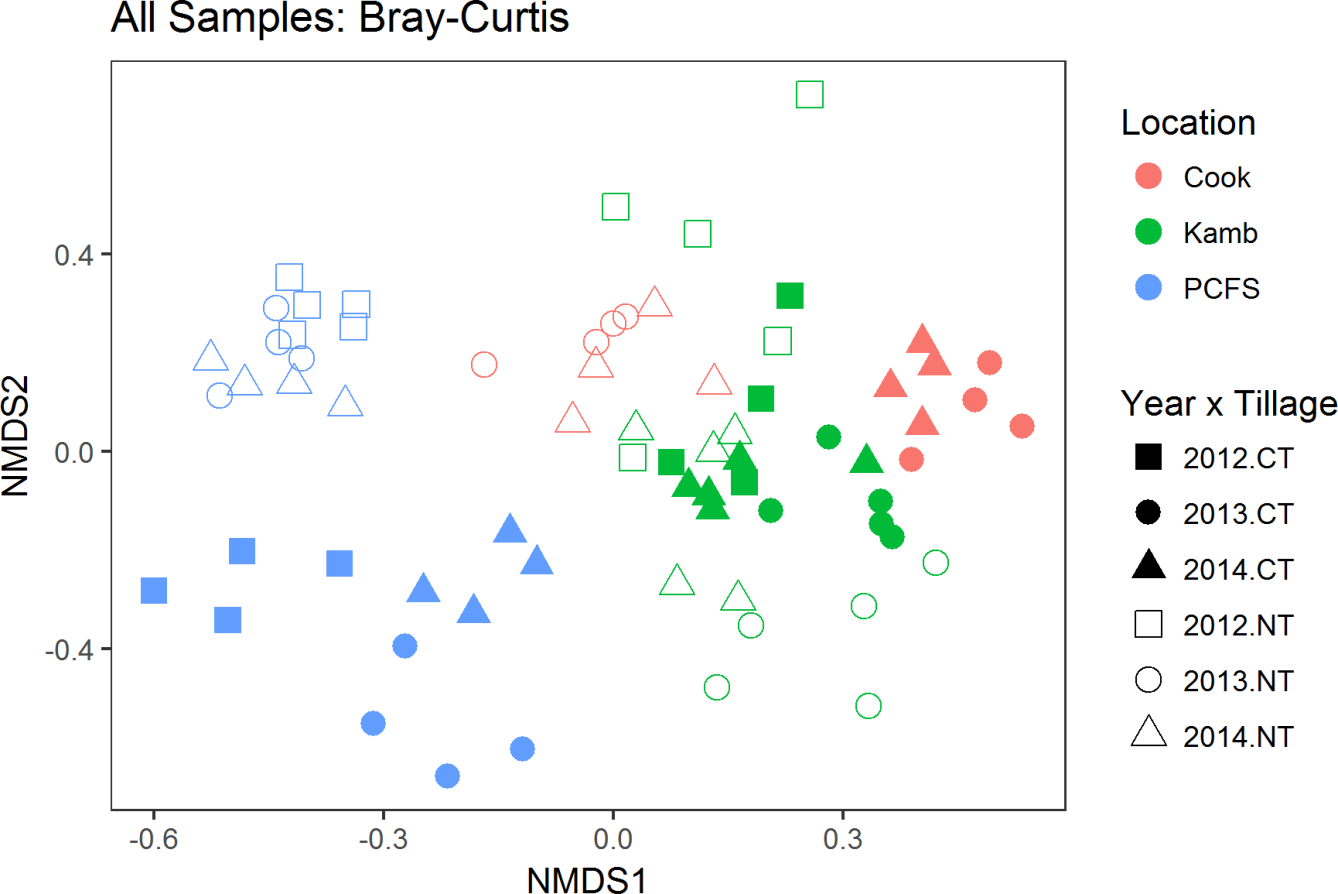
NMDS plot of fungal communities colored by location (red = Cook Farm, green = Kambitsch Farm, blue = Palouse Conservation farm). Different symbols indicate sampling year and tillage treatment (CT = conventional tillage, NT = no-till).

**Fig 2:**
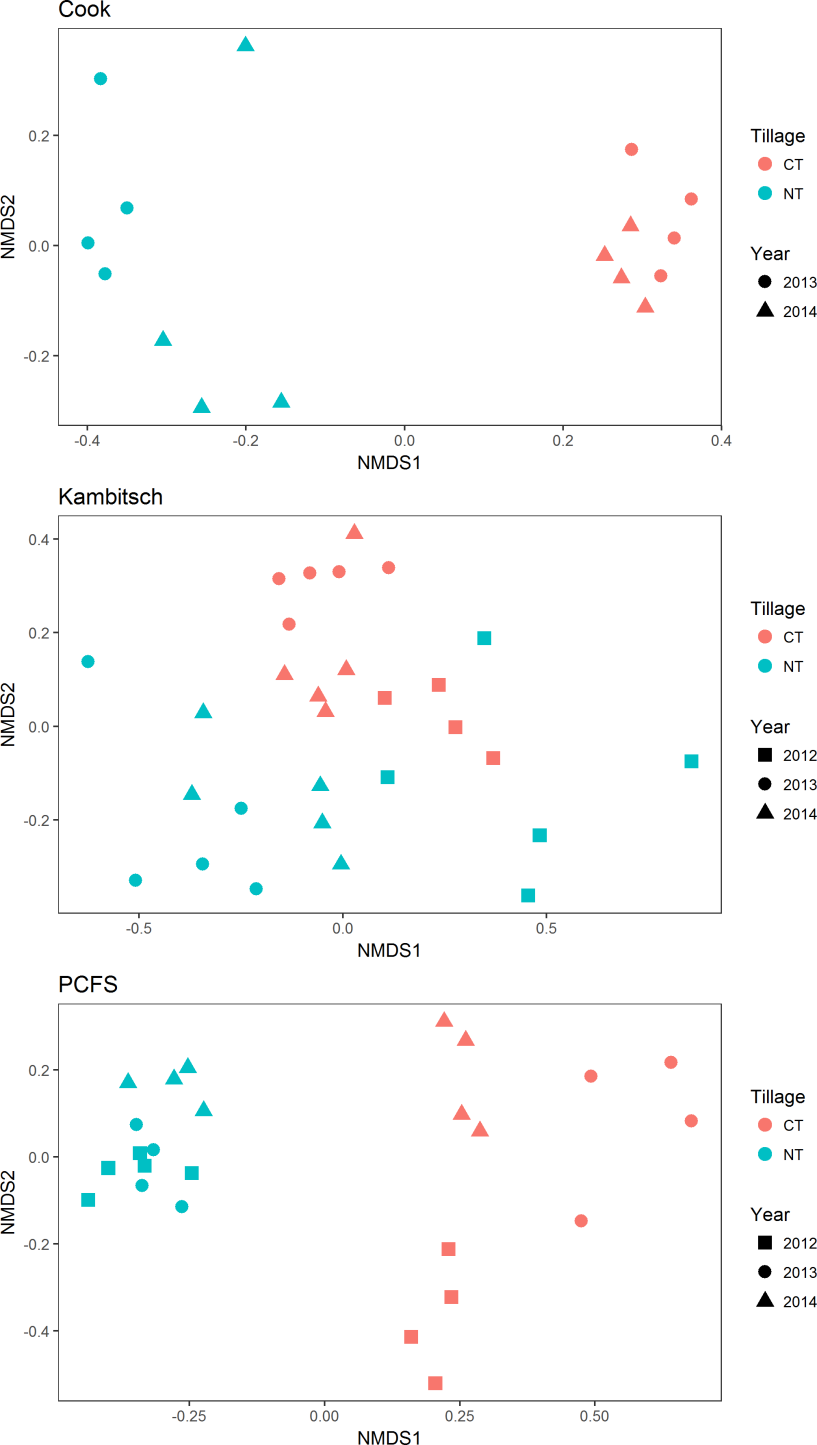
NMDS plots of fungal communities within locations colored by tillage treatment (red = conventional tillage, CT; blue = no-till, NT). Symbols indicate different sampling years.

Fungal communities varied significantly among locations, tillage treatments, and years (Fig 1, Table 1). Overall, location explained the largest amount of variation in fungal community structure (r^2^ = 0.17), followed by sampling year (r^2^ = 0.09) and tillage treatment (r^2^ = 0.08). However, within each location, fungal communities from NT fields were consistently distinct from those CT fields, regardless of the year in which they were sampled (Fig 2; Table 2). Within each location there was also significant year-to-year variation in fungal community structure in both CT and NT fields (Table 2). Interestingly, the relative importance of tillage treatment and year in determining fungal community structure varied among locations (Table 2). Notably, tillage had a much weaker influence on fungal communities at the Kambitsch Farm than the other two locations. The Kambitsch Farm had a shorter history of no-till than the other sites. There were also significant tillage × location and tillage × year interactions (Table 2).

**Table 1:** Factors determining fungal community structure as assessed by PERMANOVA.

Factor	F-value	r^2^	p-value
Tillage	9.55	0.08	**0.001**
Location	10.99	0.17	**0.001**
Year	5.76	0.09	**0.001**
Tillage x Location	5.05	0.08	**0.001**
Tillage x Year	1.91	0.03	**0.003**
Location x Year	2.86	0.07	**0.001**
Tillage x Location x Year	2.14	0.05	**0.001**

**Table 2:** Effects of tillage and year on soil fungal communities within each location as assessed with PERMANOVA.

Location	Factor	F-value	r^2^	p-value
**Cook**	Year	2.17	0.098	**0.031**
	Tillage	5.57	0.252	**0.001**
	Year x Tillage	2.35	0.106	**0.014**
**Kambitsch**	Year	3.77	0.208	**0.001**
	Tillage	3.23	0.089	**0.001**
	Year x Tillage	1.2	0.066	0.164
**PCFS**	Year	5.71	0.228	**0.001**
	Tillage	13.17	0.263	**0.001**
	Year x Tillage	3.26	0.13	**0.002**

### Fungal community diversity metrics

**Fig 3:**
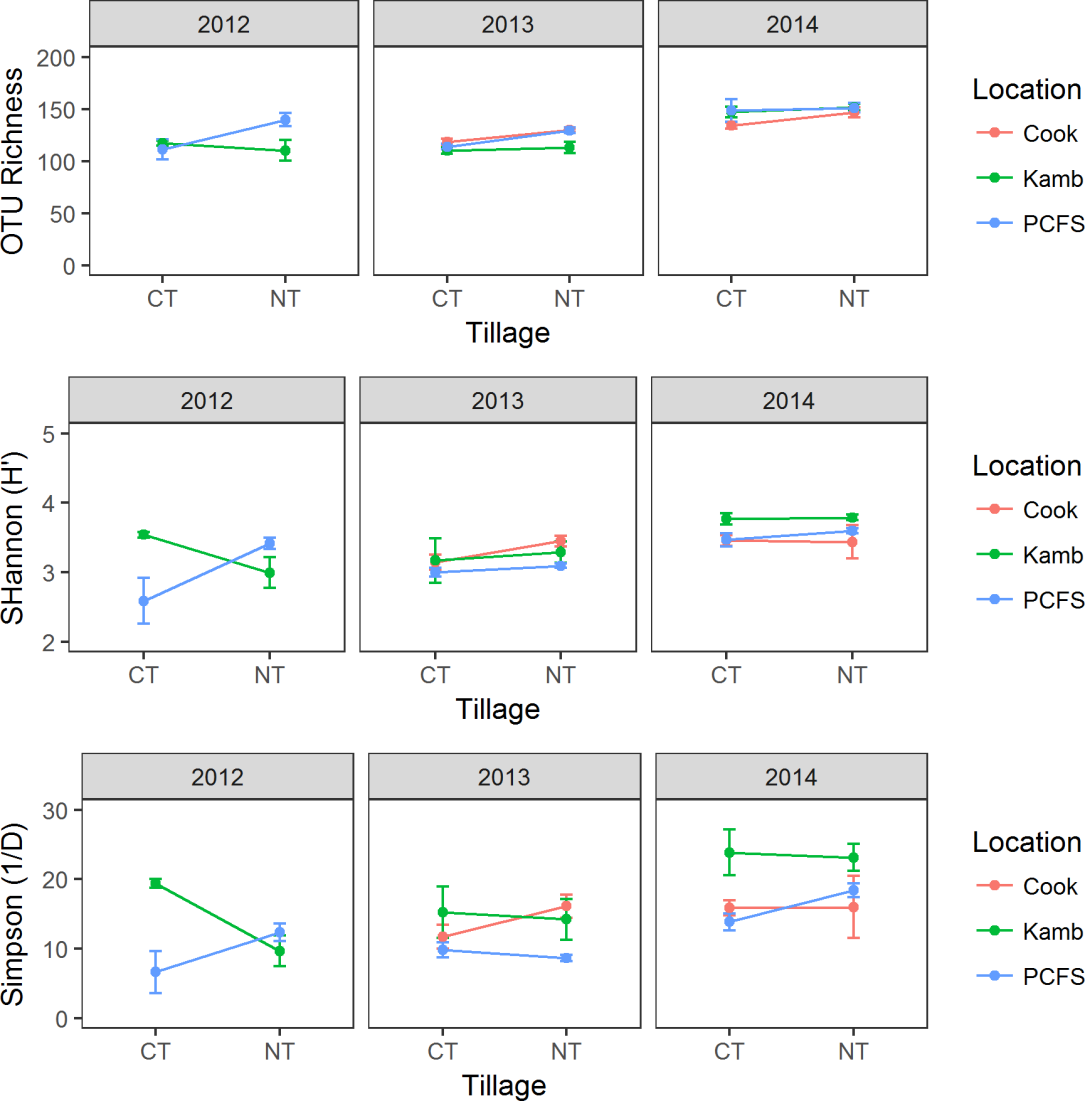
OTU richness (A), Shannon diversity (H’; B), and inverse Simpson’s diversity (1/D; C) between tillage treatments in each year and location.

Despite the large effect of tillage on fungal community structure within each location, fungal richness and diversity was most strongly related to the year of sampling (Table 3, Fig 3). In general, communities sampled in 2014 had greater fungal richness and diversity than those from 2013 or 2012, suggesting that yearly (or seasonal) environmental variation has a significant impact on fungal diversity. Tillage treatment was a significant factor determining fungal richness (p = 0.02), where fungal communities from NT fields tended to have a greater number of OTUs than those from CT fields for the PCFS and Cook Farm. However, this was not the case for the Kambitsch Farm and individual contrasts between tillage treatments were not statistically significant (data not shown). In contrast to the PCFS or Cook farms, the Kambitsch location tended to have greater diversity when assessed by the inverse Simpson’s index, suggesting greater evenness of fungal communities under CT at this location.

**Table 3:** ANOVA for fungal richness, inverse Simpson's diversity, and Shannon diversity indices among tillage treatments, farm locations, and sampling years.

	Richness (no. OTUs)		Inverse Simpson's (1/D)		Shannon Index (S')	
Factor	F-value	p-value	F-value	p-value	F-value	p-value
Tillage	5.73	**0.02**	0	0.98	1.43	0.24
Location	2.23	0.117	8.31	**0.0007**	2.6	0.083
Year	45.95	**<0.0001**	20.78	**<0.0001**	19.15	**<0.0001**
Tillage x Location	2.25	0.11	3.2	**0.048**	3.1	0.053
Tillage x Year	0.49	0.45	0.57	0.45	0.29	0.59
Location x Year	1.05	0.36	0.62	0.54	0.2	0.82
Tillage x Location x Year	1.94	0.15	1.64	0.2	3.62	**0.033**

### Variation in fungal genera among tillage practices

**Fig 4:**
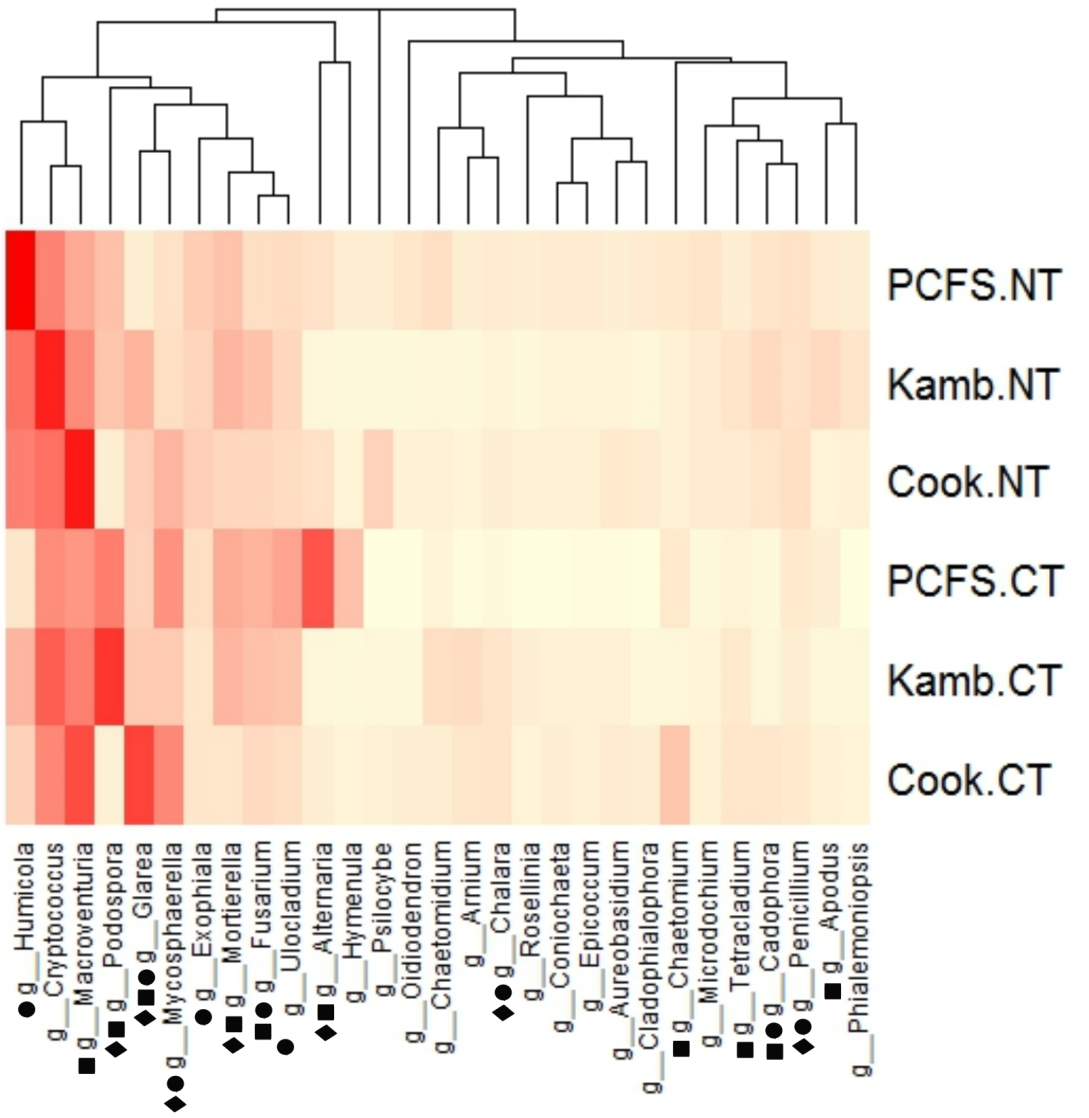
Heatmap of relative abundances of genera representing >0.5% sequences of classified taxa among tillage treatments from different locations. Shapes by genera names indicate significant differences among tillage treatments (circles), locations (squares), or years (diamonds) as assessed by ANOVA with log transformed values.

The relative abundances of fungal genera often varied significantly among tillage treatments, locations, and years (Fig 4; S1 Table). For example, fungal communities from fields under long-term no-till management consistently had higher proportions of *Exophiala* and *Humicola* than communities from conventionally tilled fields. For some genera (eg. *Cadophora*), relative abundances were greater in communities from no-till systems in some locations (Kambitsch and PCFS), but not others (Cook), suggesting that location may influence the response of some fungal genera to long-term no-till practices. In contrast to no-till, some genera had greater relative abundance in communities from conventionally tilled fields. These included *Chalara*, *Glarea*, *Mycosphaerella*, and *Ulocladium*. However, patterns in *Glarea* and *Ulocladium* were not present in all locations, again suggesting some location-specific impacts of tillage practices, as indicated by significant location × tillage interactions (S1 Table). Other abundant genera, such as *Cryptococcus* and *Macroventuria*, were equally abundant across all tillage types. At the taxonomic rank of family, fungal groups also frequently differed among tillage treatments, locations, and years (data not shown).

### Variation in individual fungal OTUs

Many fungal OTUs were identified that were significantly more or less abundant between tillage treatments (Table 4, S2 Table; n = 41 OTUs with FDR p-value <0.05), locations (n = 24 OTUs), and years (n = 10 OTUs). There were also significant tillage x location interactions with n = 15 OTUs. OTUs identified as *Humicola nigrescens*, *Cryptococcus terreus*, *Hydnodontaceae* spp., and *Exophiala* were among the most abundant that had greater relative abundances in fungal communities from no-till fields (Table 4, S2 Table), though other less abundant groups, such as *Microdochium bolleyi*, may also play important roles in these communities. Abundant OTUs that were more frequent in conventionally tilled soil communities included representatives of *Chalara*, *Mortierella*, and *Coniochaetales* spp., as well as those identified as *Cryptococcus bhutanensis*, *Chaetomium perlucidum*, and *Ulocladium chartarum* (Table 4, S2 Table). In general, patterns in the relative abundances of OTUs between tillage treatments were consistent among locations, though in some cases OTUs only differed in two of the three locations. For example, *Hydnodontaceae sp* (SH175275.07FU_GU055572) was of higher relative abundance in no-till soils at the Cook and PCFS locations, but almost absent from Kambitsch soils. Similarly, the OTU identified as *Cryptococcus bhutanensis* had a greater relative abundance in conventionally tilled soils at the Cook and Kambitsch location, but not at PCFS. Conversely, *Cryptococcus terreus* was more abundant in no-till in the Cook and PCFS, but not Kambitsch. Thus, although most fungal OTUs respond consistently to tillage practices, populations of others may be more strongly controlled by location-specific factors, such as soil characteristics or local species interactions.

**Table 4:** OTUs significantly influenced by tillage (ANOVA FDR adjusted p-value <0.05), along with ANOVA p-values for location and year effects.

Taxonomy | OTU identifier^a^	Till	Yr	Loc	Till × Yr	Till × Loc	Yr x Loc	Till × Loc × Yr
*Humicola nigrescens* SH374010.07FU_AY706334_refs	0+	0.079	1	1	0+	1	1
*Glarea lozoyensis* SH198390.07FU_FJ005111_reps	0+	0+	0.001	1	0.020	1	0.109
*Mycosphaerella tassiana SH216250*.*07FU_EF679363_refs*	0.007	0+	1	1	1	0.155	1
*Cryptococcus terreus* SH357827.07FU_AF444351_refs	0+	0.642	0.426	1	0.009	1	1
*Ulocladium chartarum* SH216785.07FU_AF229488_refs	0.028	1	1	1	1	1	1
Helotiales sp. SH204310.07FU_JX974734_reps	0.001	0+	0+	1	1	1	0.137
Tremellomycetes sp. SH190741.07FU_HG532069_reps	0+	1	0.008	1	0+	1	1
Helotiales sp. New.ReferenceOTU20	0.016	0+	0.016	1	1	0.779	1
Hypocreales sp. SH175275.07FU_GU055572_reps	0.003	0.642	1	1	0.218	1	1
*Mortierella* sp. SH180134.07FU_KF428242_reps	0+	0.135	0+	1	1	0.105	1
Hydnodontaceae sp. SH186054.07FU_HQ212160_reps	0+	1	0.021	1	0.031	1	1
Coniochaetales sp. SH011282.07FU_KC965268_reps_singleton	0.019	1	0.397	1	1	1	1
*Mortierella rishikesha* SH180109.07FU_HQ630308_refs	0.013	0+	0.835	1	0.442	0.416	1
Incertae sedis sp. SH408326.07FU_FJ427063_refs	0+	1	0+	1	1	1	1
*Cryptococcus bhutanensis* SH278429.07FU_AF145317_refs	0+	1	0+	1	0.003	1	1
*Chalara* sp. SH204486.07FU_AY969323_reps	0+	0.058	0.924	1	1	1	1
*Chaetomium perlucidum* SH195314.07FU_HQ607856_reps	0+	1	0.001	0.204	0+	1	1
*Microdochium bolleyi* SH213512.07FU_KF646098_reps	0.031	0.013	1	1	1	1	1
*Tetracladium* sp. SH020300.07FU_KC966090_reps_singleton	0+	1	0+	1	0.218	1	0.928
Coniochaetales sp. New.ReferenceOTU4	0.001	1	1	1	1	1	1
*Hymenula cerealis* SH186776.07FU_HQ322364_refs	0.004	1	0+	1	0+	1	0.946
*Phialemoniopsis curvata* New.CleanUp.ReferenceOTU2	0.002	0.303	0+	1	1	1	1
*Chaetomidium gallecicum* SH195324.07FU_JN573175_reps	0+	0.076	0+	1	0+	1	0.037
Fungi sp. SH008253.07FU_FR871193_reps_singleton	0+	0.047	0+	1	0.642	1	1
Ascomycota sp. SH185508.07FU_KC007266_reps	0+	0.023	1	1	0.030	1	1
Lasiosphaeriaceae sp. New.ReferenceOTU5	0+	0.817	0+	1	0+	0.005	0.008
*Cryptococcus* sp. New.ReferenceOTU12	0+	1	0+	1	0+	1	1
*Penicillium novae-zeelandiae* SH407703.07FU_JN617688_refs	0+	1	1	1	1	1	1
Trechisporales sp. SH188947.07FU_JF691365_reps	0+	1	0+	1	0+	1	1

0+, value <0.001. OTU identifiers correspond to representative sequences in the UNITE v.7 dynamic (31.01.16) reference set.

### Plant pathogenic genera and OTUs

One of the most common genera of wheat pathogens is *Fusarium* (*Fusarium pseudograminearum* and *F*. *culmorum*), the cause of Fusarium crown rot. We identified 8 OTUs classified as *Fusarium* and 8 in the family Nectriaceae, with very abundant sequences, but because of the conserved nature of ITS sequences in this genus, they could not be identified to species. Another group of pathogens is *Rhizoctonia solani* AG-8 and other *Ceratobasidium* species, which cause root rots and would be classified in the family Ceratobasidiaceae. We identified 2 OTUs in this family, which were not very abundant. We identified one OTU of *Hymenula cerealis* (*Cephalosporium graminis*), causal agent of Cephalosporium stripe, a wilt disease. This was only found at PCFS CT, a field which was previously inoculated with the pathogen as part of a variety screening site. We identified one OTU of *Microdochium nivale*, cause of a snow mold, but this taxa was also rare. An OTU of *Microdochium bolleyi* was also identified, which was most abundant in no-till. This is a common root parasite, but is considered weakly virulent or non-pathogenic.

## Discussion

This work demonstrates that tillage practices have a profound impact on soil fungal communities in agricultural systems. Though a few recent studies have used next-generation sequencing to look at how tillage affects the soil microbiome, most of these have investigated other components of the soil community, such as bacteria or arbuscular mycorrhizal fungi. For example, Yin et al. [26] described bacterial communities at the same locations used in this work, and showed that tillage had rather minor effects on overall community structure, compared to location and proximity to the root (bulk soil vs rhizosphere). We hypothesized that fungal communities would be more strongly influenced, because of the crucial role that fungi play in residue decomposition, compared to bacteria.

Degrune et al. [32] found tillage significantly affected fungal communities, but that tillage was less important than soil depth. Unlike most previous work with high-throughput sequencing, they found that fungal diversity and richness declined with reduced tillage compared to conventional tillage, and that several taxa present in conventional tillage were lost in the reduced tillage treatment. Our findings were contrary to this, and fit most previous work in finding higher abundance, richness and diversity with no-till [15, 17, 33]. Although we did not specifically look at AMF, most of the literature, including recent work with high-throughput sequences, has confirmed the detrimental effects of tillage on AMF. Detheridge et al. [34] found that tillage had no major effect on fungal communities, except that AMF were more abundant in no-till and pathogenic fungi were more abundant in plowed soils.

One of the most obvious interactions in wheat cropping systems between fungi and tillage concerns plant pathogenic fungi that rot roots and crowns of wheat. There is abundant literature on how no-till or reduced tillage may increase fungal diseases of wheat, especially those that survive in crop residue. For example, Fusarium head blight caused by *Fusarium graminearum* (*Gibberella zeae*) is increased when residue is left on the surface, because the pathogen can overwinter and produce fruiting bodies and ascospores on the straw. Although it is typically too dry for Fusarium head blight in the dryland PNW, Fusarium crown rot caused by *F*. *pseudograminarum* and *F*. *culmorum* are widespread and major yield reducers [35, 36]. Some studies have shown increased crown rot under no-till [37], but we find it also in conventional systems. Unfortunately, the sequencing of the fungal ITS region does not provide sufficient resolution to identify *Fusarium* species. Sequencing with the translation elongation factor 1 alpha is the standard for identifying *Fusarium*. However, we identified 8 OTUs of *Fusarium* and 8 OTUs belonging to Nectriaceae, the family that contains *Fusarium*. Interestingly, *Fusarium* were one of most abundant genera overall, but showed no trends with tillage treatments. From other surveys in the world [38], at least a dozen species can colonize wheat straw, including *F*. *equisiti*, *F*. *acuminatum*, and *F*. *avenaceum*. Additional sequencing using alternative targets specific to *Fusarium* (eg. *RPB2*; [39]) may reveal interesting patterns in this genus with tillage. Another group that is affected by tillage is *Rhizoctonia solani* AG-8, which increases when tillage is stopped [37]. We detected two OTUs in the Ceratobasidiaceae, but the abundances were low and no trends were observed. However, we were successful in identifying two other potential pathogen groups at the species level. *Microdochium nivale*, cause of a snow mold, was in low abundance and showed no trends. However, *Microdochium bolleyi* was of relatively high abundance, and increased in the no-till treatments across all locations and years. This taxon is considered to be a weak parasite of wheat roots [40], a potential biocontrol agent against other root pathogens [41] and has been shown in another study to be more common in no-till, based on isolation from wheat roots [42]. We also detected *Hymenula cerealis*, also known as *Cephalosporium gramineum*. This causes a wilt disease, and was only found in high levels in the PCFS CT site. This was the site of a previous Cephalosporium stripe nursery that had been inoculated with the pathogen in previous years.

There were a few groups of saprophytic fungi that were more predominant in no-till. One was *Humicola nigrescens*, (Family Chaetomiaceae), a dematiaceous fungus. *Humicola* contains 20 species, mostly isolated from soil and plant tissue [43]. Many species are thermophilic [44]. It is closely related to *Trichocladium*. *T*. *aspergum* was found to be the dominant late colonizer of rye straw [45] and a dominant member of the soil cellulotyic community [46]. *Exophiala* was also more predominant in no-till. Like *Humicola*, this is a dark mycelial, dark-spored genus is found in soil, leaf litter, and wood. Some species are also pathogens of mammals, amphibians and fish [43]. *Cadophora* spp. are dark-septate root endophytes. Some are pathogens of soybean [47], grape [48] and tree species such as willow [49]. Some are also considered potential biocontrol agents [50] and may benefit tree health [51].

More groups of fungi were more abundant in the conventionally tilled treatment. One of the most abundant was *Mycosphaerella tassiana*, the perfect stage of *Cladosporium herbarum*. It is also classified as *Davidiella* (Family Mycosphaerellaceae). *Cladosporium* are extremely common in soil, plant surfaces and are excellent colonizers of necrotic plant tissue. They produce abundant spores and are often found in air samples, both indoor and outdoor. *Ulocladium* (Family Pleosporaceae) occupies a similar niche, with abundant spores, and were also more abundant in conventionally tilled soils. Two OTUs in the order Coniochaetales were higher in conventional tillage. This group in the class Sordariomycetates forms perithecia, and are found on wood, bark and the soil [52].

One unusual finding was the genus *Glarea*. First described in the late 1990s, the most widely studied species is *G*. *lozoyensis*, which produces a novel antifungal compound pneumocandin, with applications in medicine [53]. The most abundant *Glarea* OTU showed 99% similarity with *G*. *lozoyensis*. It was more abundant in conventional tillage, and was found at all three locations. Another genus found more abundant in conventional tillage was *Chalara* (Family Helotiateae). This is related to *Thielaviopsis basicola*, which is a pathogen of rotation crops such as pea and causes black root rot.

But a larger number of genera did not show any general trend with tillage, although they were very abundant. This included *Fusarium* and *Mortierella*. However, individual OTUs within a genus may show a trend, for example a *Mortierella* sp. OTU which was more common in conventional tillage. *Mortierella* is in the Division Zygomycota and is a very common soil fungus and root colonizer. Also common were *Penicillium* and *Aspergillus* (Family Trichocomaceae), common cellulolytic colonizers of soil and plant residue. The most unusual finding was *Macroventuria* (Family Didymellaceae). The taxonomic status of this was recently resolved [54] and one OTU showed 97.5% similarity to *M*. *anomochaeta*, originally isolated from decaying canvas in South Africa and sequenced [54]. It probably has cellulolytic capability, but may be part of the *Didymella-Phoma* complex which also contains pathogens of rotation crops such as peas and chickpeas. As with all identifications based on a limited sequence, a caveat needs to be made that only by isolating and characterizing the isolate, can one be completely sure of species identification. In addition, there can be PCR primer bias that can favor certain groups. But nevertheless, *Macroventuria* was a very abundant taxon.

Although Ascomycota was the predominant phylum, there were a few groups of basidiomycetes that were common and more predominant in no-till. We detected 2 OTUs in the family Hydnodontaceae and one in the order Trechisporales. The order Trechisporales contains one family, Hydnodontaceae, with 15 genera [52]. They produce a macroscopic thallus, a corticioid, recupinate flat structure, with a hymenium covered with basidia and basidiospores. There is not much literature on this group, but like most basidiomycetes in the Agaricales, they are prolific producers of enzymes that break down lignins and wood. The genus *Trechispora* has a wide spread distribution in forest ecosystem, but little is known about corticioid fungi in agricultural soils. Lynch and Thorn [55] examined a clone library from DNA extracted from an agricultural soil in Michigan. They sequenced the large and small subunit RNA and identified 215 homobasidiomycetes species, including the order Ceratobasidiales (*Rhizoctonia*, *Waitea*) and Tremellales (*Cryptococcus*), but none in the order Trechosporiales.

The most abundant Basidiomycete group was the genus *Cryptococcus* (Family Tremellaceae), a very common soil inhabitant worldwide, with the vegetative form of a single-celled yeast [56]. It is found in forest and grassland soils based on DNA sequences [57–59]. *Cryptococcus* is a colonizer of wheat roots [60] and strawberry roots [61]. It also contains the human pathogens *C*. *neoformans* and *C*. *gatti* [62]. These are able to survive dry soil conditions by the formation of a polysaccharide capsule and melanin [62]. We identified 4 species that were highly abundant, *C*. *terreus* (more abundant in no-till), and *C*. *victoriae*, *C*. *aerius* and *C*. *bhutanesis* (more abundant in conventional tillage). This group of fungi is probably not capable of degrading complex plant structural components, like the homobasidiomycetes, but is perhaps using simpler components of breakdown products [63]. A number of them have been shown to be effective biocontrol agents [63] and may play a role in the suppression of root diseases.

After an initial increase in wheat root diseases, especially Rhizoctonia, disease often declines in long-term no-till systems [20, 22]. The buildup of populations of pathogen-antagonistic fungi has been implicated as the responsible agents for disease suppression [24]. As such, the taxa found as more abundant in no-till soils may be good candidates for exploration as biocontrol agents. However, targeted culturing approaches will be needed to isolate and investigate the pathogen-antagonistic potential and other functional characteristics of these taxa. Similarly, because amplicon sequencing provides only relative abundance data, complementary quantitative approaches, such as qPCR, will offer additional insight into the absolute abundances of these groups and their importance in soil communities in different cropping systems.

In conclusion, the fungal community in wheat soils is highly influenced by the tillage system. This effect was evident, despite the fact that location and year of sampling also had major effects. Even though we sampled the winter wheat part of the rotation in all treatments, the previous rotations were different, which could explain some of the variation. We identified a consortia of taxa in high abundance that were more dominant in no-till and one that was more dominant in conventional tillage, consistent across locations and years. Our results suggest that taxa more dominant in no-till are better adapted at utilizing intact, decaying roots as a food source and may exist as root endophytes. This would give them a competitive advantage in colonizing the dying root. However, another possibility is that these populations are negatively impacted by tillage, which can break up hyphal networks or cause modify other components of the soil microbiome, leading to a secondary effects on fungi. Our results suggest that taxa more common in conventionally tilled systems can utilize fresh, mature plant residues that are turned into the soil with tillage as pioneer colonizers, and then produce large numbers of conidia that are not as affected by tillage as the mycelial life stage. However, a larger portion of the community also showed no significant difference in abundance in both systems. Taxa unaffected by tillage treatments may be niche generalists, survive in deeper soil layers so as to avoid negative effects, or may be less affected by the bacterial microflora which can be stimulated by tillage.

## Supporting information

S1 Fig Phylum-level composition of no-till (NT) and conventionally tilled (CT) fields in different locations and years. (TIF)

S1 Table FDR corrected p-values for ANOVAs on Log10 transformed sequence counts comparing tillage, location, and year effects on abundant fungal genera. Mean sequence counts and standard deviations are presented for each location/tillage combination.
(DOCX)

S2 Table OTUs significantly influenced by tillage (ANOVA FDR adjusted p-value <0.05). Mean sequence counts (± standard deviation) for each tillage-location combination are presented.
(DOCX)

S3 Table Rarefied OTU table. (XLSX)

S4 Table OTU representative sequences. (FNA)
