# Supplementary figures and images for: Long-term no-till: A major driver of fungal communities in dryland wheat cropping systems
Source: PLoS One. 2017 Sep 12;12(9):e0184611. doi: 10.1371/journal.pone.0184611 (PMC5595340; doi:10.1371/journal.pone.0184611)

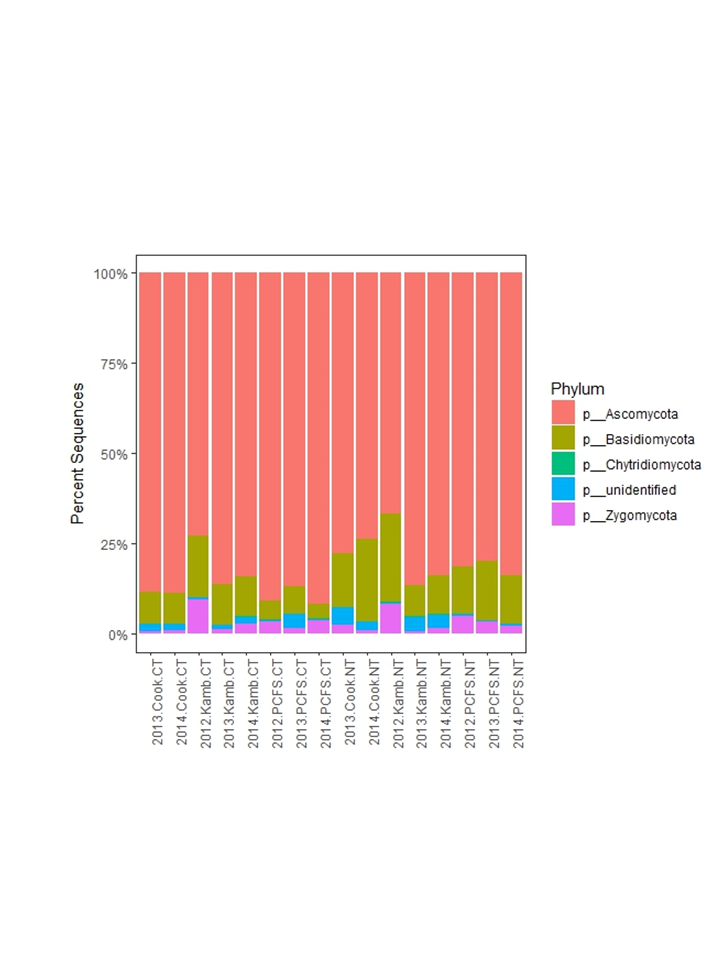

Supplement: S1 Fig — (TIF) [file pone.0184611.s001.tif]
