# Supplementary material for: Long-term no-till: A major driver of fungal communities in dryland wheat cropping systems
Source: PLoS One. 2017 Sep 12;12(9):e0184611. doi: 10.1371/journal.pone.0184611 (PMC5595340; doi:10.1371/journal.pone.0184611)
Supplement: S1 Table — Mean sequence counts and standard deviations are presented for each location/tillage combination. (DOCX) [file pone.0184611.s002.docx]

Supplemental Table 1. FDR corrected p-values for ANOVAs on Log10 transformed sequence counts comparing tillage, location, and year effects on abundant fungal genera. Mean sequence counts and standard deviations are presented for each location/tillage combination

| **Fungal Genera** | **Till** | **Loc** | **Yr** | **Till × Yr** | **Till × Loc** | **Yr × Loc** | **Till × Yr × Loc** | **Cook-CT** | **Cook-NT** | **Kamb-CT** | **Kamb-NT** | **PCFS-CT** | **PCFS-NT** |
| --- | --- | --- | --- | --- | --- | --- | --- | --- | --- | --- | --- | --- | --- |
| *Alternaria* | 1.000 | **0.000** | **0.000** | 1.000 | 0.336 | **0.000** | **0.000** | 7.6 **±** 6.5 | 39 **±** 71.3 | 4.6 **±** 4.5 | 6.4 **±** 8.1 | 217.8 **±** 349.7 | 37.7 **±** 49.9 |
| *Apodus* | 0.994 | **0.020** | 0.570 | **0.028** | 0.457 | 1.000 | 1.000 | 0.3 **±** 0.7 | 2.3 **±** 2.9 | 4.5 **±** 5 | 61.5 **±** 128.9 | 20.4 **±** 24.8 | 8.2 **±** 9.4 |
| *Cadophora* | **0.023** | **0.008** | 0.170 | 1.000 | 1.000 | **0.011** | 0.169 | 28.3 **±** 25.6 | 29.4 **±** 16.2 | 5.6 **±** 6.5 | 57.7 **±** 182.1 | 7 **±** 5.1 | 27.2 **±** 29.6 |
| *Chaetomidium* | 0.082 | 0.083 | **0.017** | 1.000 | 0.000 | **0.008** | 0.488 | 10.8 **±** 9.4 | 5.9 **±** 6.8 | 62.6 **±** 63.8 | 3 **±** 2.6 | 13.3 **±** 19.4 | 46.1 **±** 35.6 |
| *Chaetomium* | **0.002** | **0.000** | 0.131 | **0.004** | 0.000 | **0.001** | 1.000 | 120 **±** 49.2 | 8 **±** 13.6 | 8.4 **±** 6.5 | 16.3 **±** 32.3 | 28.2 **±** 42.9 | 17.4 **±** 11.1 |
| *Chalara* | **0.000** | 0.144 | **0.008** | 1.000 | 1.000 | 1.000 | 1.000 | 38.8 **±** 39.6 | 9.5 **±** 16.2 | 51.6 **±** 92.3 | 9.4 **±** 21.6 | 8.8 **±** 9.8 | 5.6 **±** 18.2 |
| *Cryptococcus* | 0.385 | 0.083 | **0.049** | 1.000 | **0.015** | 0.079 | 1.000 | 280.9 **±** 82.5 | 288.9 **±**132.2 | 358.3 **±** 161 | 416.2 **±** 522 | 145.8 **±** 67.7 | 327.8 **±** 117.5 |
| *Exophiala* | **0.047** | 0.431 | 0.373 | 1.000 | 0.447 | 0.435 | 1.000 | 27.6 **±** 15.5 | 81 **±** 33.4 | 44.1 **±** 29.2 | 67.7 **±** 71.3 | 39.9 **±** 30.5 | 102.5 **±** 58.2 |
| *Fusarium* | 0.104 | 0.166 | 0.570 | 1.000 | 1.000 | 1.000 | 1.000 | 63.5 **±** 26.3 | 64.5 **±** 78.3 | 135.4 **±** 102 | 104.3 **±** 97.1 | 96.8 **±** 48.8 | 45 **±** 20.4 |
| *Glarea* | **0.000** | **0.000** | **0.000** | 1.000 | **0.003** | **0.094** | **0.016** | 465.6 **±** 308.9 | 83.3 **±** 210.3 | 107.2 **±** 156.5 | 134.4 **±** 193.8 | 58.3 **±** 118.2 | 2.1 **±** 4.3 |
| *Humicola* | **0.000** | 0.431 | **0.010** | 0.138 | 0.000 | 1.000 | 1.000 | 84.1 **±** 39.1 | 254.8 **±** 120 | 153.7 **±** 85.8 | 256.9 **±** 197.2 | 32.3 **±** 35.9 | 730.9 **±** 356.2 |
| *Macroventuria* | 1.000 | **0.000** | 0.570 | 1.000 | 0.143 | 1.000 | 1.000 | 435.5 **±** 184.2 | 485.1 **±**231.7 | 280 **±** 136.1 | 211.6 **±** 138.5 | 131.9 **±** 64.7 | 208.5 **±** 75.4 |
| *Mortierella* | 1.000 | 0.157 | **0.000** | 1.000 | **0.023** | 0.093 | 1.000 | 27.1 **±** 9 | 61.9 **±** 38.7 | 155.9 **±** 142.9 | 131.1 **±** 298.2 | 107.1 **±** 106.9 | 132.1 **±** 75.9 |
| *Mycosphaerella* | **0.001** | 0.431 | **0.000** | 0.267 | 1.000 | **0.043** | 0.282 | 284.4 **±** 241.5 | 135.5 **±** 155 | 108.4 **±** 123.4 | 50.5 **±** 54.5 | 139.2 **±** 164.2 | 39.5 **±** 67 |
| *Penicillium* | **0.007** | **0.015** | **0.000** | 1.000 | 1.000 | 0.093 | 1.000 | 25 **±** 4.3 | 47.5 **±** 21.7 | 23.3 **±** 18.2 | 46.5 **±** 39.1 | 27.7 **±** 24.3 | 36.5 **±** 24.9 |
| *Podospora* | 0.749 | **0.000** | **0.001** | 1.000 | **0.034** | **0.020** | 1.000 | 3.6 **±** 5.8 | 11.5 **±** 9.3 | 454.3 **±** 676.3 | 99.5 **±** 158.4 | 166.8 **±** 239.3 | 141.8 **±** 224.6 |
| *Tetracladium* | **0.010** | **0.000** | 0.140 | 1.000 | 0.143 | 1.000 | 0.230 | 28.8 **±** 18.5 | 18.9 **±** 26.1 | 38 **±** 37.9 | 40.7 **±** 34.6 | 13.5 **±** 12.5 | 1.1 **±** 1.1 |
| *Ulocladium* | **0.004** | 0.431 | 0.281 | 1.000 | 1.000 | 1.000 | 1.000 | 53.3 **±** 20.9 | 50.8 **±** 52.8 | 119.4 **±** 90.3 | 66.9 **±** 64.9 | 118.3 **±** 68.7 | 50.8 **±** 27.1 |
| Unidentified | 1.000 | 0.097 | **0.020** | 0.111 | **0.022** | 0.097 | 1.000 | 1544.6 **±** 435.3 | 1740 **±** 583 | 1308.4 **±** 331.2 | 1673.3 **±** 530.7 | 2105.1 **±** 517.8 | 1520.2 **±** 327.4 |
