# Supplementary material for: Long-term no-till: A major driver of fungal communities in dryland wheat cropping systems
Source: PLoS One. 2017 Sep 12;12(9):e0184611. doi: 10.1371/journal.pone.0184611 (PMC5595340; doi:10.1371/journal.pone.0184611)
Supplement: S2 Table — Mean sequence counts (± standard deviation) for each tillage-location combination are presented. (DOCX) [file pone.0184611.s003.docx]

**Supplemental Table 2. OTUs significantly influenced by tillage (ANOVA FDR adjusted p-val <0.05). Mean sequence counts (± standard deviation) for each tillage-location combination are presented.**

| **Taxonomy \| OTU identifier** | **Cook-CT** | **Cook-NT** | **Kamb-CT** | **Kamb-NT** | **PCFS-CT** | **PCFS-NT** |
| --- | --- | --- | --- | --- | --- | --- |
| *Humicola nigrescens* SH374010.07FU_AY706334_refs | 84 **±** 39 | 254 **±** 120 | 153 **±** 85 | 256**±** 197 | 32 **±** 36 | 729 **±** 355 |
| *Glarea lozoyensis*  SH198390.07FU_FJ005111_reps | 466 **±** 309 | 83 **±** 210 | 107 **±** 156 | 134 **±** 194 | 58 **±** 118 | 2 **±** 4 |
| *Mycosphaerella tassiana* *SH216250.07FU_EF679363_refs* | 284 **±** 241 | 134 **±** 154 | 108.**±** 123 | 50 **±** 54 | 137 **±** 162 | 39 **±** 67 |
| *Cryptococcus terreus*  SH357827.07FU_AF444351_refs | 41 **±** 20 | 110 **±** 73 | 68 **±** 32 | 80 **±** 83 | 55 **±** 31 | 190 **±** 84 |
| *Ulocladium chartarum*  SH216785.07FU_AF229488_refs | 52 **±** 21 | 49 **±** 51 | 118 **±** 89 | 66 **±** 64 | 116 **±** 68 | 50 **±** 26 |
| Helotiales sp.  SH204310.07FU_JX974734_reps | 273 **±** 177 | 43 **±** 45 | 80 **±** 59 | 59 **±** 57 | 24 **±** 43 | 2**±** 5 |
| Tremellomycetes sp.  SH190741.07FU_HG532069_reps | 25 **±** 11 | 73 **±** 54 | 80 **±** 29 | 74 **±** 43 | 25 **±** 10 | 82 **±** 34 |
| Helotiales sp.  New.ReferenceOTU20 | 10 **±** 9 | 54 **±** 59 | 15 **±** 21 | 199 **±** 251 | 24 **±** 79 | 12 **±** 33 |
| Hypocreales sp.  SH175275.07FU_GU055572_reps | 14 **±** 5 | 75 **±** 59 | 67 **±** 66 | 67 **±** 59 | 19 **±** 8 | 58 **±** 36 |
| *Mortierella* sp.  SH180134.07FU_KF428242_reps | 12 +/- 7 | 1 **±** 2 | 111**±** 125 | 30 **±** 42 | 18 **±** 15 | 1.**±**- 2 |
| Hydnodontaceae sp.  SH186054.07FU_HQ212160_reps | 0 **±** 0 | 202 **±** 499 | 0.1 **±** 0.3 | 0 **±** 0 | 0 **±** 0 | 48 **±** 100 |
| Coniochaetales sp.  SH011282.07FU_KC965268_reps_singleton | 22**±** 13. | 8**±** 7 | 51**±** 32 | 11 **±** 14 | 47**±** 59 | 25 **±** 16 |
| *Mortierella rishikesha*  SH180109.07FU_HQ630308_refs | 7 **±** 4 | 23 **±** 21 | 22 **±** 23 | 22 **±** 27 | 13 **±** 8 | 67 **±** 48 |
| Incertae sedis sp.  SH408326.07FU_FJ427063_refs | 15 **±** 7 | 13 **±** 23 | 66 **±** 37 | 30 **±** 23 | 4 **±** 4 | 0.2 **±** 0.8 |
| *Cryptococcus bhutanensis*  SH278429.07FU_AF145317_refs | 73**±**14 | 16 **±** 20 | 44 **±** 23 | 11 **±** 8 | 5 **±** 5 | 4 **±** 23 |
| *Chalara* sp.  SH204486.07FU_AY969323_reps | 39 **±** 40 | 9 **±** 16 | 52 **±** 92 | 9 **±** 22 | 9 **±** 10 | 6 **±** 18 |
| *Chaetomium perlucidum*  SH195314.07FU_HQ607856_reps | 101 **±** 43 | 3 **±** 3 | 6 **±** 5 | 10 **±** 21 | 14 **±** 11 | 7 **±** 7 |
| *Microdochium bolleyi*  SH213512.07FU_KF646098_reps | 4 +/- 4 | 18 **±** 21 | 12 **±** 10 | 26 **±** 56 | 9 **±** 15 | 32 **±** 27 |
| *Tetracladium* sp. SH020300.07FU_KC966090_reps_singleton | 29 **±** 18 | 1 **±** 2 | 28 **±** 37 | 25**±** 35 | 8 **±** 9 | 0.4 **±** 0.7 |
| Coniochaetales sp. New.ReferenceOTU4 | 13 **±** 9 | 6 **±** 7 | 28 **±** 15 | 11 **±** 16 | 21 **±** 14 | 7 **±** 4 |
| *Hymenula cerealis* SH186776.07FU_HQ322364_refs | 0 **±** 0 | 0 **±** 0 | 0.1 **±** 0.3 | 0 **±** 0 | 79 **±** 238 | 0.2 **±** 0.6 |
| *Phialemoniopsis curvata* New.CleanUp.ReferenceOTU2 | 0 **±** 0 | 9 **±** 24 | 7 **±** 23 | 41 **±** 85 | 0.2 **±** 0.4 | 1 **±** 2 |
| *Chaetomidium gallecicum* SH195324.07FU_JN573175_reps | 6 **±** 6 | 0.4 **±** 0.5 | 44 **±** 50 | 1**±** 1 | 1 **±** 3 | 5 **±** 7 |
| Fungi sp. SH008253.07FU_FR871193_reps_singleton | 3 **±** 5 | 33 **±** 42 | 0.5 **±** 1 | 18 **±** 28 | 0 **±** 0 | 0.2 **±** 0.6 |
| Ascomycota sp. SH185508.07FU_KC007266_reps | 0.8 **±**1 | 14 **±** 9 | 6 **±** 5 | 11 **±** 12 | 1 **±** 3 | 12 **±** 11 |
| Lasiosphaeriaceae sp. New.ReferenceOTU5 | 0 **±** 0 | 0 **±** 0 | 0 **±** 0 | 0 **±** 0 | 0 **±** 0 | 41 **±** 73 |
| *Cryptococcus* sp. New.ReferenceOTU12 | 2 **±** 2 | 21.**±** 10 | 3 **±** 4 | 1 **±** 2 | 2 **±** 2 | 15 **±** 7 |
| *Penicillium novae-zeelandiae* SH407703.07FU_JN617688_refs | 6 **±** 6 | 11 **±** 7 | 2 **±** 2 | 8 **±** 8 | 3 **±** 4 | 11 **±** 7 |
| Trechisporales sp. SH188947.07FU_JF691365_reps | 0.4 **±** 0.7 | 24 **±** 18 | 0 **±** 0 | 0.2 **±** 0.8 | 0 **±** 0 | 20 **±** 22 |

0+, value <0.001
